# Myc proteins in brain tumor development and maintenance

**DOI:** 10.3109/03009734.2012.658975

**Published:** 2012-04-19

**Authors:** Fredrik J. Swartling

**Affiliations:** Uppsala University, Department of Immunology, Genetics, and Pathology, Rudbeck Laboratory, SE-751 85 Uppsala, Sweden

**Keywords:** Brain tumors, molecular biology, oncogenes, pediatrics, protein phosphorylation, tumor biology

## Abstract

Myc proteins are often deregulated in human brain tumors, especially in embryonal tumors that affect children. Many observations have shown how alterations of these pleiotropic Myc transcription factors provide initiation, maintenance, or progression of tumors. This review will focus on the role of Myc family members (particularly c-myc and Mycn) in tumors like medulloblastoma and glioma and will further discuss how to target stabilization of these proteins for future brain tumor therapies.

## Introduction

The Myc family comprises the transcription factors c-myc, Mycn, and Mycl (1). Myc proteins are important for normal development, especially *c-myc* and *Mycn* which are embryonically lethal when knocked out (2-4) in transgenic mice. Transgenic mice present a severely diminished forebrain and hindbrain when *Mycn* is specifically abrogated in nestin-positive neural stem and precursor cells (NSCs) of the developing brain (5), while a similar conditional *c-myc* knock-out moderately impairs brain growth (6). Nestin-driven transgenes for double *c-myc* and *Mycn* depletion have a nervous system phenotype that is much more severe than either Myc gene knock-out alone (7,8). These findings collectively suggest that Myc proteins are essential for brain development. Overexpression of c-myc in nestin-positive NSCs promotes cell proliferation (9), and many human tumors including brain tumors express high levels of or show gene amplifications of Myc family members. This is true for medulloblastoma (10–12) and glioma (13–15), the most common malignant types of brain tumors in children and adults, respectively. It is also true for other brain tumor types, like primitive neuroectodermal tumors (PNETs) (16). Similarly, overexpressed c-myc or Mycn have been found to initiate different types of brain tumors like medulloblastoma (17–20), PNETs (21), and glioma (22,23) in mice. In most of these cases Myc genes generate tumors after a rather long latency or in combination with other oncogenes (like Ras, Akt, Shh, or beta-catenin) or tumor suppressor genes (e.g. p53, Ptc, Ink4c). This suggests a necessary involvement of one or more additional transforming events before a full-blown cancer can develop. It is not known in detail how Myc proteins use other cancer genes to induce these tumors and if there is a difference in how the different Myc proteins are involved in brain tumor initiation. This is also difficult to study as Myc genes target a large number of other genes and regulate important cellular mechanisms like proliferation, apoptosis, DNA repair, metabolism, ribosome biogenesis, and protein synthesis (24). Clearly, Myc proteins can use different strategies in their role as master regulators of cell proliferation and in tumor maintenance.

## Medulloblastoma biology

Brain tumors represent the most common solid tumor of childhood. The embryonal tumor medulloblastoma (MB) is next to leukemia the most common malignancy in children (25). Treatment includes surgery, radiation, and chemotherapy, which cures about 70% (26), although survivors can have severe long-term side-effects following this treatment. MB can be categorized into different subtypes (27) presenting a desmoplastic or nodular, classic, and large cell/anaplastic (LCA) pathology, where the LCA subtype shows monomorphic cells with large nuclei and correlates with the poorest outcome. Advanced genomic and transcriptional profiling has recently offered reliable molecular classification of human MB (28–31). This provides a better classification of these childhood tumors, which will facilitate better strategies for improved patient treatment. Abnormal activation of molecular pathways like Sonic Hedgehog (SHH) and Wingless (WNT) signaling contributes to tumorigenesis in some patients; however, the majority show no abnormalities in these pathways and are referred to as Group 3 or Group 4 MB (32). Wnt signaling abnormalities involving beta-catenin nuclear staining occur in 10%–15% of patients and have been described as a marker for favorable outcome (33,34). Such tumors have recently been found to originate from BLBP-positive cells of the lower rhombic lip structure in the developing dorsal brain stem (35). Another developmental pathway believed to have a critical role in MB maintenance is Notch. Inhibiting Notch suppresses MB *in vivo* by apoptosis and may prove effective in MB therapy (36,37), even if Notch is not required in Shh-dependent MB subtypes (38,39). Shh-dependent MB only represents about 25% of MB cases. Nevertheless there are numerous reports of Shh-dependent models that recapitulate human Shh-dependent MB in mice (see recent review (40)); the first were models that activated the pathway through loss of Patched (41). Amplification of *MYC* or *MYCN* occurs in about 10% of human MB and correlates with a 5-year overall survival of only 13% (11). Both *MYC* and *MYCN* amplifications further associate with an aggressive LCA medulloblastoma pathology (10,42,43). *MYCN* is expressed at high levels in SHH-associated MB (10) and is actually essential for Shh-dependent tumors in mouse models of this disease (17). However, most Shh-independent human MB also express *MYCN* (20,44,45). Indeed, in a model where human *MYCN* drives MB formation from the glutamate transporter 1 (Glt1) promoter in cerebellar cells, the majority of the developed brain tumors are actually Shh-independent (20). This MB model thus correlates with recent findings (34,46) that suggest a majority of *MYCN*-amplified human MB are categorized as non-SHH Group 4 MB (46).

## Glioma biology

Glioma, the most common primary brain tumors in adults, is classified as grade I to IV according to the World Health Organization (WHO) (47). Of high-grade gliomas (grade III and IV), glioblastoma (GBM) is the most common and most malignant (grade IV) tumor with dismal outcome. GBMs account for 60%–70% of malignant glioma (48). While grade III gliomas (anaplastic astrocytoma, anaplastic oligodendroglioma, and anaplastic oligoastrocytoma) are characterized by increased cellularity, nuclear atypia, and proliferative activity, GBMs also contain areas of microvascular proliferation and necrosis. GBMs have a 5-year overall survival of less than 10% despite an increased survival effect from the use of the alkylating agent temozolomide following tumor resection and radiation (49). An effort to stratify GBMs further and to enable a more individualized therapy is further to classify these malignant brain tumors into different subtypes characterized by molecular abnormalities (50–52). The most recent efforts of genomic profiling from The Cancer Genome Atlas (TCGA) Research Network have defined four subgroups of GBM: proneural, neural, classical, and mesenchymal. These subgroups show signature aberrations where the gene expression of EGFR, NF1, and PDGFRA/IDH1 can help define a classical, mesenchymal, and proneural subtype, respectively (52). There are many reports in which Myc proteins have been amplified or overexpressed in glioma (53–56). Interestingly, *MYCN* showed high-level focal amplifications in a subset of GBM samples (51), and *MYC* or *MYCN* is found to be amplified in almost half of brain cancers that have combined features of malignant glioma and primitive neuroectodermal tumors (MG-PNET) (15). Interestingly, p53, which is directly or indirectly inactivated in 87% of GBMs (TCGA), represses *MYC* transcription by directly binding to the *MYC* promoter (57). Most important, c-myc plays a critical role in the regulation and especially the proliferation of glioma stem cells, which are the putative cells of origin for these brain tumors (58-60). This suggests that Myc proteins are master regulators also for these types of brain tumors.

## Myc signaling in normal brain

**Figure 1.**
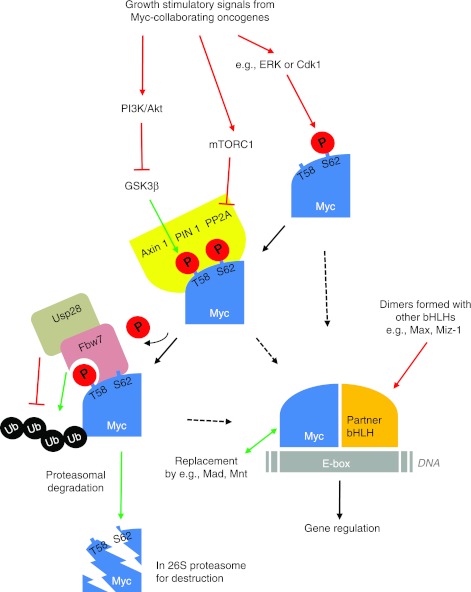
Schematic model of the regulatory pathways for Myc proteins from activating signals of collaborating oncogenes to protein destabilization and proteasomal degradation. Suggested activating pathways (in red) and inhibiting pathways (in green) with putative involvement in brain tumor development and maintenance.

Myc proteins are members of a large family of basic helix-loop-helix (bHLH) transcription factors. These protein members can form homo- or heterodimeric complexes with themselves or with other members of this family. One important prerequisite for Myc activity is its interaction with a dimerization partner, the bHLH protein Max (Figure 1) (61). Such generated Myc-Max complexes bind to E-box sequences which are found in promoters of many genes that act to promote transcription and cell proliferation (62,63). Through Miz1-complexes Myc and Max have been shown to have repressive functions (64) inhibiting tumor suppressor genes/cell cycle regulators like p15Ink4b and also p21Cip1 (65), where Myc represses p21Cip1 expression via a Miz1-dependent interaction with the p21 promoter (66). Moreover, the partners Mad or Mnt (another bHLH protein) act in Max-dimeric complexes transcriptionally to repress genes associated with E-box sequences (67).

Myc members are differently expressed in normal brain. Mycl has no reported phenotype in knock-out mice but has particularly high expression in the ventricular zone of the embryonic midbrain (8,68). Mycn is highly expressed in developing forebrain and hindbrain (5), while c-myc is expressed at lower levels (6,8). In normal cerebellar granule cells, Mycn is important for cell replication, whereas Mycn protein degradation is essential for cell cycle exit (69–71). Importantly, Mycn depletion in nestin-driven NSCs reduces both germinal zones of the developing cerebellum, including the neuroepithelial cells lining the fourth ventricle as well as the rhombic lip structure with the granule precursor cells forming the external germinal layer (5). The depletion also leads to disrupted neuronal differentiation which correlates with the findings that Myc genes (including c-myc, Mycn, and Mycl) promote (but are not required for (72)) the dedifferentiation processes for producing induced pluripotent stem (iPS) cells (73,74). High expression of Myc proteins, at least when using v-myc (the retroviral homolog of c-myc) transduction, is important for immortalization and self-renewal of NSCs (75,76). Interestingly, Mad proteins also appear to have a role in neuronal differentiation (77). Mxi1, another member of the Mad family of proteins, can antagonize Myc oncoproteins *in vivo* (78). Overexpression of Mxi1 in human glioma cell lines reduces their mitotic activity (79), possibly through down-regulation of cyclin B1 expression (80). The data suggest that Mxi1 that interacts with Max can antagonize Myc and thereby suppress glioma progression.

## Myc protein stability

Myc proteins are extremely unstable with half-lives of only 20–30 min (81). There are two phosphorylation sites on Myc proteins which are primarily responsible for stabilization (Figure 1). First, serine (S62) is phosphorylated which leads to Myc stabilization (82). The extracellular-regulated kinase 1, 2 (ERK) is known to mediate S62 phosphorylation (83). However, in cerebellar neural precursors, the mitotic cyclin-dependent kinase 1 (Cdk1) is the priming kinase for Mycn at S62 (70). Following S62 phosphorylation of Myc, threonine 58 (T58) is phosphorylated by glycogen synthase kinase 3 beta (GSK3β) which will target Myc for degradation (84). Under normal conditions T58 is always phosphorylated after S62.

The turnover of Myc is mostly controlled by ubiquitin-mediated proteolysis (85,86), which targets the specific Myc proteins with great precision. Specific ubiquitin-protein ligases (of type E3) like Fbw7 can recognize and bind to Myc, but only when T58 is phosphorylated alone. This requires dephosphorylation of S62 with the help of a prolyl isomerase, Pin1, that binds the phosphorylated sites and isomerizes Myc on proline 59 (87). This provides a conformation change of Myc which enables protein phosphatase 2A (PP2A) to remove the phosphate group. Fbw7 can now recognize Myc with a single phosphorylation on T58 and send Myc for proteolytic degradation (88) by ubiquitylating Myc on its amino-terminus. It is suggested that at least four ubiquitin groups need to be linked to a substrate in order for it to be recognized by a proteasome that will later dissect it into small peptide fragments (89).

Increased stability of Myc proteins can explain the reported inconsistency between gene amplifications and protein levels of Myc found in solid tumors, like in breast cancer (90,91). There are numerous reports of enhanced expression levels of Myc oncoproteins in many different types of tumors, that suggesting this is an important event for tumor initiation or an apparent advantage for tumor progression (reviewed in (92)). Myc protein levels are elevated in subtypes of glioma (14), but there are few reports in which a correlation between expression/amplification and protein levels of Myc proteins is assessed. Such a relationship would be required in order to understand fully the extent of putative Myc protein stabilization in these brain tumors.

Reports of mutations or alterations in genes that can regulate Myc protein stability are increasing (for a recent review, see (93)). For example, Fbw7 can keep Myc levels low and therefore act as a tumor suppressor. In the absence of Fbw7, the levels of Myc and the activity of the protein will increase (94), and mutations in Fbw7 prevent Myc degradation in T-cell acute lymphoblastic leukemia (T-ALL) (95). With regard to the discussions of how brain tumor develops it is interesting to find that Fbw7 is highly expressed during normal brain development (96,97), where it can possibly regulate Myc protein stability. Other ubiquitin ligases recognize Myc on other sites than Fbw7 does, like S phase kinase-associated protein 2 (Skp2). Skp2 is responsible for Myc protein turnover (98) but is paradoxically also promoting Myc activity by functionally amplifying the Myc response (99). How Skp2 is working like an oncogene and how it correlates with Myc is not fully known (93), but Skp2 is actually associating with Myc target genes when Myc is active (100). Other proteins involved in regulating stability of Myc proteins are also mutated in cancer, like Usp28, which has been found frequently up-regulated in colon adenocarcinomas (101). This soluble deubiquitylating enzyme (DUB) stabilizes Myc by removing linked ubiquitin chains that have been conjugated by Fbw7 (Figure 1). TRIM32 is another ubiquitin ligase that is asymmetrically distributed during NSC division and is inhibiting cell proliferation by promoting Myc degradation and neuronal differentiation (102). However, whether TRIM32 can also regulate proliferation by differentiating brain tumor cells is currently not known.

## Myc mutants

A mutation causing a switch from threonine to alanine on residue 58 was discovered in the transforming v-myc viral gene and in Myc or Mycn in Burkitt's lymphoma patients (103,104). This mutation prevents the phosphorylation from GSK3β that is so important for Fbw7 recognition which can lead to proteolytic degradation of Myc proteins. When searching the Catalogue of Somatic Mutations In Cancer (COSMIC) (105), very few mutations for Myc genes are found in the category of central nervous system (CNS) tumors (Table I). Here, only one mutation in *MYC* and two (with one being a silent mutation) in *MYCN* were reported (from The Cancer Genome Atlas (TCGA) genotyping screen (51)). Whether any of the non-silent mutations (Table I) could affect Myc protein function or stability has not been reported.

**Table I.** Few mutations of Myc genes are found in brain tumor samples^a^.

Gene^b^	AA mutation	CDS mutation	Sample name	Sample ID	Histology (WHO grade)
*MYC*	p.R316R	c.948G > A	TCGA-02-0083	1287236	Glioblastoma (IV)
*MYCN*	p.P365P	c.1095A > G	TCGA-02-0010	1287210	Glioblastoma (IV)
*MYCN*	p.P44L	c.131C > T	TCGA-02-0028	1287216	Glioblastoma (IV)

^a^The mutation data were obtained from the Sanger Institute Catalogue of Somatic Mutations in Cancer web site (as of October, 2011), http://www.sanger.ac.uk/cosmic (105), in the Primary Tissue category of Central Nervous System (CNS) tumors.

^b^For MYC, one mutation was found in 524 cases examined in the category of CNS tumors. For MYCN, two mutations were found in 469 cases examined in the category of CNS tumors. No mutations were reported for MYCL1 in the category of CNS tumors (45 cases). WHO = World Health Organization; AA = amino acid; CDS = coding sequence; TCGA = The Cancer Genome Atlas (51).

## Myc and Ras collaborate to transform cells

Expression of c-myc together with a co-operative oncogene like Ras is necessary for the stable transformation of primary or early-passage fibroblasts (106). Ras can stabilize Myc proteins in tumors. One example is through high ERK-mediated Myc S62 phosphorylation in Ras-transformed cells (83). Different activating mutations of Ras promote ERK-mediated phosphorylation and are implicated in brain tumors, but it is important to distinguish if and how the different isoforms collaborate with Myc proteins in brain tumor formation and maintenance. For example, H-ras^G12V^ can collaborate with v-myc to generate brain tumors in human fetal neural stem cells (23), but H-ras^Q61L^ cannot help v-myc to generate brain tumors in human adult neural stem cells in another report (107). It is possible that fetal neural stem cells are more prone to transformation than adult neural stem cells or that the different activating mutations have different effects on brain tumor development. Myc proteins might require different isoforms of Ras proteins as collaborators in brain tumorigenesis. For example, when searching COSMIC (105) there are reports of mutations in CNS tumors (like glioma and PNETs) only for N-ras (8/1017) and K-ras (8/1054), but not for H-ras (108). Mutations found in K-ras and N-ras were at residues 12 or 61. For N-ras it is evident that all mutations at residue 12 (4/1017) were found only in glioma samples, while mutations at residue 61 (4/1017) were found only in PNET or medulloblastoma. Interestingly, in the Myc-Ras collaboration, Myc represses the cellular senescence induced by Ras. This repression of senescence by Myc requires phosphorylation of Myc at S62 by the cell cycle kinase Cdk2 that is needed for inhibiting cellular senescence induced by c-myc (109,110). Many oncogenes like Ras (22,23), Akt (22), Shh (17), or beta-catenin (21) can be involved in collaborating with Myc proteins to induce brain tumors. This property of Myc proteins to launch collaborations during tumor formation warrants more high-throughput screens in order to identify critical Myc collaborating genes using previously successful strategies with retroviral tagging (111,112) or Sleeping Beauty techniques (113–116) in mice.

## How can we inhibit Myc proteins in brain tumors?

Many tumors show addiction to Myc oncoproteins (117). This is also true in Mycn-driven medulloblastoma models in which inhibition of Mycn will result in total tumor regression and cellular senescence (20). In glioma where suppressor genes like p53 and Pten are inactivated, c-myc is essential for tumor maintenance, and c-myc inhibition will suppress tumors by promoting differentiation of the glioma cells (60). It is evident that Myc proteins are validated targets for cancer therapies (as reviewed in (118,119)). Targeting these transcription factors that lack clear binding domains have always proved difficult, and using short interfering RNAs (like specific short hairpin RNAs) that target Myc directly is not yet a treatment option for patients. There are, however, small molecular drugs that inhibit Myc-Max interactions that are effective in cancers like human acute myeloid leukemia (120-122). Promoting Mad, another bHLH protein that antagonizes Myc, would be another promising approach in brain tumor therapy (123).

There are other ways to induce growth arrest and senescence in childhood medulloblastoma and in atypical teratoid/rhabdoid tumor cells by using G-quadruplex interactive agents in order to disable c-myc at the promoter level (124). It is also possible to target regulatory components in which Myc controls ribosome biogenesis (125). Myc can regulate transcription of ribosomal proteins through RNA polymerase II (RNA pol II) (126,127). MYC participates in release of paused RNA pol II, as c-myc can bind positive elongation factor b (P-TEFb) and stimulate transcriptional elongation in cancer cells. Combined targeting of c-myc and P-TEFb could prove effective for tumors maintained by Myc proteins (128). Another strategy is to force changes in chromatin modification controlled by Myc genes (129). For example, histone lysine side-chain acetylation increased by c-myc can be effectively suppressed by inhibition of acetyl-lysine recognition domains (bromodomains) in multiple myeloma, a Myc-dependent hematologic cancer (130).

Pathways downstream of receptor tyrosine kinases like MAPK/ERK and PI3K/Akt/mTOR that indirectly control Myc protein stability (Figure 1) are often overexpressed or altered in brain tumors. MAPK/ERK kinase inhibitors can dephosphorylate c-myc and reduce cell proliferation and anchorage-independent growth of rhabdomyosarcoma (131), a soft tissue sarcoma in children. However, downstream of Shh signaling, Cdk1 rather than ERK is associated with S62 activity, at least in granule neuron precursors (69,70) that could serve as medulloblastoma cells of origin (13,132). The cyclin-dependent kinase sibling Cdk2 can also phosphorylate S62. Yet, while Cdk2 inhibitors will promote senescence in Myc-induced cells (133), Cdk1 inhibitors (134) can be used to promote the apoptotic effects induced by Myc proteins (135). This is also the suggested cell death that temozolomide treatment promotes from c-myc via Akt signaling in O6-methylguanine-DNA methyl transferase (MGMT) expressing glioblastoma (136). Akt activity also determines the sensitivity to mammalian target of rapamycin (mTOR) inhibitors by regulating c-myc expression (137). Moreover, mTOR exists in a complex, mTORC1, that directly phosphorylates and inhibits PP2A (138) which (as described above and visualized in Figure 1) will lead to sustained Myc protein activity. Consequently, clinical inhibitors of PI3K/mTOR prove efficacy when used to degrade Mycn in neuroblastoma (139,140), a childhood tumor thought to originate from the peripheral neural crest. Such PI3K/mTOR inhibitors are indeed also effective in suppressing glioma (141) and medulloblastoma (142). Other examples to target Myc protein stability include treatment with clinically available synthetic steroid drugs, like dexamethasone, that can be used to destabilize Mycn, leading to inhibited growth of Shh-dependent medulloblastoma (143). Ultimately, drugs that can target regulators of the ubiquitin-proteasome system (reviewed in (144)) can promote final degradation of long-lived and harmful Myc proteins also in brain tumor cells. To summarize, there are numerous observations about how Myc proteins co-ordinate cell transformation and many promising ideas on how to target these proteins in brain tumors, so let's keep on hunting.
